# Does α ^+^-Thalassaemia Protect against Malaria?

**DOI:** 10.1371/journal.pmed.0030235

**Published:** 2006-05-09

**Authors:** Geoffrey Pasvol

## Abstract

Pasvol discusses a new study in *PLoS Medicine* that shows that α ^+^-thalassemia protects against severe but not mild malaria.

Ever since Haldane proposed in 1949 that thalassaemia might protect individuals against the scourge of malaria [ 1], the challenge has been to provide supporting evidence—be it at the cellular, clinical, or epidemiological level. The general topic of human red cell polymorphisms and malarial protection has attracted enormous interest, largely because this subject provides the most compelling example of natural selection and, hence, “survival of the fittest” in humans. Sickle cell trait remains the paradigm of a red cell polymorphism that protects against malaria. Several lines of evidence, including plausible cellular mechanisms, confirm that this haemoglobinopathy provides up to 90% protection against death due to malaria [ 2]. However, there are many gaps in our knowledge of whether other common red cell variants, notably thalassaemia, might protect against malaria and of the mechanism of such protection.

## Interactions between Thalassaemias and Malaria

Why has investigation of the interaction between thalassaemias and malaria remained inconclusive despite our best efforts? For ß-thalassaemia, it is only in a limited part of West Africa that it continues to co-exist with malaria. Malaria has been eradicated from most other regions where ß-thalassaemia is common. The findings of Willcox et al. support a protective role for ß-thalassaemia, in which heterozygotes one-to-four years old appear to have a reduced risk of malaria, using an arbitrary density criterion of 1 × 10 ^9^/l relative to controls (relative risk, 0.45; upper 95% confidence interval, 0.79) [ 3]. By contrast, α ^+^-thalassaemia (in which one or two of the four α-globin genes are deleted) is exceedingly common throughout sub-Saharan Africa, an area of high malarial transmission. However, thus far, the effects of α ^+^-thalassaemia on the clinical manifestations of malaria in this area have not been extensively studied. And the few studies that have been carried out on the relationship between α ^+^-thalassaemia and malaria have not provided an all-encompassing and plausible cellular mechanism for protection.

There are many gaps in our knowledge of whether thalassaemia might protect against malaria.

Malarial parasites can certainly invade and multiply within a ^+^-thalassaemic cells [ 4]. They also appear to cytoadhere equally well to endothelial cells as do parasitized normal red cells [ 5]. So how does thalassaemia protect against malaria? Carlson and colleagues proposed (with supporting evidence) that thalassaemic cells have a reduced ability to form rosettes (a process in which uninfected red cells bind to infected cells), which causes harm perhaps by aiding and abetting the obstruction of capillary blood flow and leading to sequestration of schizont-infected red cells in vital organs [ 6]. Thalassaemic red cells also appear to bind increased amounts of immunoglobulin, which might favour early removal of those red cells containing parasites [ 7]; perhaps, they bind less complement, sparing those cells with reduced complement [ 8]. We just do not know why thalassaemia protects against malaria, leaving our current knowledge in a state of conjecture. While study of the association of disease with genetic polymorphisms may be intellectually attractive, some would argue that it is of little clinical relevance.

## A New Study on α ^+^-Thalassaemia

Notwithstanding this, Sammy Wambua, Thomas Williams, and colleagues, [ 9] in a new study in *PLoS Medicine*, have now cogently approached the conundrum of a ^+^-thalassaemia and malaria in a structured and compelling fashion within an area of intensive malarial transmission in sub-Saharan Africa. They studied two cohorts of children: (1) a cohort with “mild disease”, recruited from an age-stratified population sample, which was monitored using weekly clinical surveillance for three years; and (2) a “birth cohort” of children born between 1992 and 1995 within a defined geographical area of the Kenyan coast in East Africa, whose admissions to hospital were recorded. Slide-positive parasitaemia (with or without symptoms), as well as parasite density, was clearly defined. Moreover, definitions of severe malaria—largely, coma, more than two seizures in 24 hours, parasitaemia greater than 20%, and severe malarial anaemia (haemoglobin concentration less than 50 g/l)—were carefully delineated.

In brief, the authors found that α ^+^-thalassaemia did not protect against parasitisation in asymptomatic individuals. Furthermore, α ^+^-thalassaemia did not protect against symptomatic malaria, nor did it lead to a reduction in the density of parasites in peripheral blood. In the birth cohort, there was reduced admission to hospital of α ^+^-thalassaemic children with malaria or severe malaria. An even more tantalizing finding was that thalassaemia provided no protection against cerebral malaria unless accompanied by anaemia—the protection by α ^+^-thalassaemia appeared to be mainly confined to severe anaemia. α ^+^-thalassaemia also appeared to protect against nonmalarial anaemia.

The researchers also investigated other major nonmalarial diagnoses—such as upper and lower respiratory tract infections, gastroenteritis, and skin or helminthic infections—and found that the occurrence of lower respiratory tract infections in the mild disease cohort was lower in those with α ^+^-thalassaemia. Protection by α ^+^-thalassaemia against nonmalarial disease—including respiratory infection, gastroenteritis, and meningitis—has previously been documented [ 10]. Perhaps most interesting was that although average haemoglobin concentrations were lower in the children with α ^+^-thalassaemia, both in steady state and during mild malarial attacks, this result was reversed in severe disease when the children with thalassaemia had relatively higher haemoglobin concentrations.

Could thalassaemia, in reality, protect against nonmalarial anaemia as found in this study? The absence of malaria parasites in the blood of an individual with severe anaemia living in a highly endemic area for malaria might not be sufficient to exclude a diagnosis of malaria. Malarial infection leaves a wake of red cell destruction, which often continues long after parasites have disappeared or are microscopically undetectable. The question of whether thalassaemia protects against nonmalarial anaemia can only be addressed in a study carried out in a nonmalarious area where thalassaemia and equivalent socioeconomic deprivation occur. Such an area would be hard to find: the coexistence of deprivation and malaria go almost hand in hand [ 11].

## Conclusion

This extensive and well-executed study concludes that α ^+^-thalassaemia does not protect individuals against acquiring malarial infection, becoming symptomatic, or developing high parasite densities. However, the study also concludes that the risks of developing severe malaria, especially malarial anaemia, are reduced in people with α ^+^-thalassaemia (the implication is that people with α ^+^-thalassaemia also have reduced risk of malaria-specific mortality, although the authors did not show this). How those with thalassaemia appear to be protected against developing severe anaemia with or without malaria and why they might be protected against lower respiratory tract infections remain unexplained. Perhaps answers to these questions might turn out to be more rewarding than they first appear.
